# A small solitary non-parasitic hepatic cyst causing an intra-hepatic bile duct stricture: a case report

**DOI:** 10.1186/1752-1947-4-254

**Published:** 2010-08-07

**Authors:** Keunho Lee, Taeho Hong

**Affiliations:** 1 Department of Surgery, Incheon ST. Mary's Hospital, College of Medicine, The Catholic University of Korea, Incheon, Korea; 2 Department of Surgery, Seoul ST. Mary's Hospital, College of Medicine, The Catholic University of Korea, Seoul, Korea

## Abstract

**Introduction:**

We report an unusual presentation of a small hepatic cyst causing cholangitis.

**Case presentation:**

A 70-year-old Asian man was hospitalized for aggravated chronic pain in the right upper portion of his abdomen. Fever developed after admission. Laboratory tests revealed elevated hepatobiliary enzymes, inflammatory markers and carbohydrate antigen 19-9 without hyperbilirubinemia. Ultrasound and computed tomography demonstrated dilatation of the left intra-hepatic bile ducts. Endoscopic retrograde cholangiopancreatography showed that the right intra-hepatic bile ducts were normally filled with contrast medium, but the left intra-hepatic bile ducts were not seen in the confluence. A left hepatectomy was performed because a hidden malignancy could not be excluded. The surgical findings showed no tumor around the bile duct but rather a 2 cm cyst in segment four of Couinaud's category of the liver around the hilum. The pathology report was a solitary non-parasitic hepatic cyst compressing the bile duct.

**Conclusion:**

A very small solitary hepatic cyst might cause hepatic duct stricture if it is located near the hepatic hilum, and should be considered in the differential diagnosis of a hepatic duct stricture.

## Introduction

Solitary non-parasitic hepatic cysts (SNHC) are usually asymptomatic. Only a small fraction of them are associated with symptoms such as abdominal pain, an abdominal mass, early satiety, nausea, and vomiting. These symptomatic SNHCs are usually larger than 10 cm and can cause obstructive jaundice and cholangitis because of their mass effect on the bile ducts. In this case, we present a 70-year-old man with a very small (2 cm) cyst in the hepatic hilum compressing the left hepatic duct.

## Case presentation

A 70-year-old Asian man presented to the out-patient department complaining of pain in the upper portion of his abdomen. The pain, which started seven months previously, had worsened, and our patient complained of fever and chills with the pain. However, he was never hospitalized and did not recall any prior medical evaluation for this problem. The patient denied any other systemic symptoms such as nausea, vomiting, jaundice, and weight loss. He had no significant medical history and was not taking any medication.

On physical examination, our patient's temperature was 38.3°C with a blood pressure of 129/67 mmHg, a heart rate of 89 beats per minute and a respiratory rate of 18 breaths per minute. The abdominal examination revealed localized tenderness to palpation in the right upper quadrant but no guarding, rebound, or percussion tenderness. The rest of the physical findings were unremarkable.

Laboratory investigation showed the following results: white blood cell count 16.4 × 10^9^/L with a predominance of neutrophils, aspartate aminotransferase 72 IU/L, alanine aminotransferase 137 IU/L, total bilirubin 0.7 mg/dL, direct bilirubin 0.5 mg/dL, alkaline phosphatase 405 IU/L, and gamma glutamyl transpeptidase 155 IU/L. The carbohydrate antigen 19-9 level was slightly elevated (49.1 U/mL) and the carcinoembryonic antigen level was within the normal range.

**Figure 1:**
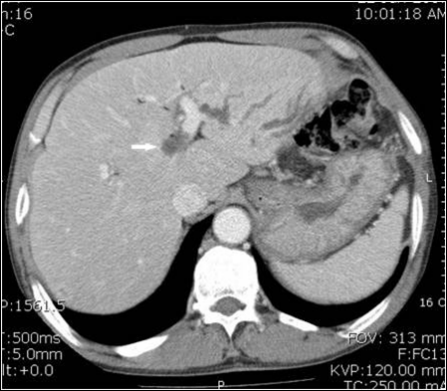
**CT**. The dilated left IHBDs are seen but otherwise it is normal. The arrow indicates a suspicious cyst on the review.

Ultrasound (US) was performed first. This showed a distended gallbladder with sludge in it and dilated intra-hepatic bile ducts (IHBD). Endoscopic retrograde cholangiopancreatography (ERCP) showed that the right IHBD normally filled with the contrast medium, but the left IHBD could not be differentiated from the confluence. We assumed that the left hepatic duct was compressed by something or that there was a stricture at this site; perhaps caused by a tumor near the hepatic hilum. An abdominal contrast-enhanced computed tomography (CT) was performed to confirm the findings. However, no abnormality was detected except for the dilated left hepatic bile ducts on the CT scans (Figure 1). An intra-operative US failed to gain any further information beyond that obtained through the pre-operative US.

**Figure 2:**
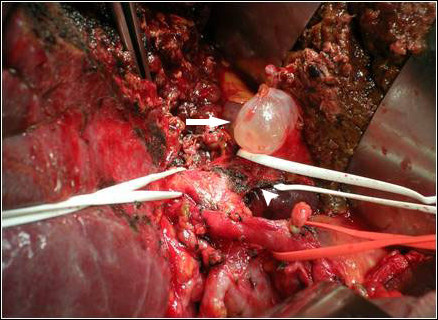
**Intra-operative photograph**. The small hepatic cyst (arrow; 1.5 × 2.0 cm in size) was near the confluence of the right and left hepatic ducts compressing the left hepatic duct (arrow head).

**Figure 3:**
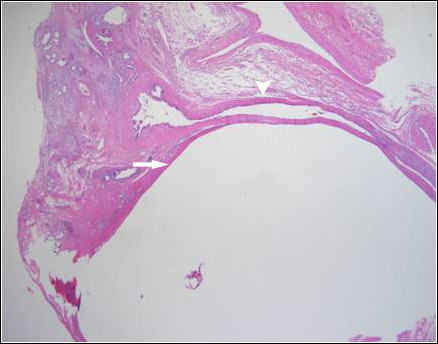
**Microscopic finding (×40)**. The hepatic cyst (arrow) compresses the hepatic duct (arrowhead) and there is no communication between the two structures.

We performed a left hepatectomy on July 11, 2007 because we could not exclude a malignancy of the left hepatic duct. There were mild adhesive changes around the liver that might have been caused by cholangitis. No tumor lesions were found around the bile duct. Only a small hepatic cyst (1.5 × 2.0 cm size) was present, in segment four according to Couinaud's classification at the level of the transverse fissure, compressing the left hepatic duct (Figure 2). It was confirmed as a solitary benign non-parasitic cyst lined by a single layer of cuboidal epithelium on histological examination (Figure 3). Our patient made an uneventful recovery and at the five-month follow-up, he was asymptomatic and all laboratory findings had normalized.

## Discussion

SNHCs are usually asymptomatic but may occasionally present with abdominal pain, an abdominal mass, early satiety, nausea, and vomiting. However, even with symptomatic hepatic cysts, obstructive jaundice or cholangitis is rarely seen. Several reports on SNHC causing obstructive jaundice are presented in the medical literature since first described by Caravati *et al. *in 1950 [1].

Most prior cases were over 10 cm and the symptoms usually resulted from a mass effect of the enlarging cyst on the neighboring bile ducts [2,3]. However, Tsuyoshi *et al. *and Matthieu *et al. *each reported very exceptional cases of 3 cm and 4 cm sized SNHCs causing dilatation of the IHBD [4,5]. Both of these small cysts were located in segment four according to Couinaud's classification, near the hepatic hilum. Our patient had a 2 cm cyst in segment four at the level of the transverse fissure and compressing the left hepatic duct. These cases demonstrate that the position, as well as the size, of the cyst is important for compression or stricture of the bile ducts.

IHBD strictures can be malignant or benign. Malignant causes are mostly cholangiocarcinoma, hepatocellular cancer and metastatic cancers to the liver. While benign causes are numerous and include recurrent pyogenic cholangitis related to hepatolithiasis (previously known as Oriental cholangiohepatitis), primary sclerosing cholangitis, radiation, blunt abdominal trauma, polyarteritis nodosa and systemic lupus erythematosus, tuberculosis and histoplasmosis, chemotherapeutic drugs infused into the hepatic artery, a choledochal cyst, cystic fibrosis with liver involvement, space occupying lesions in the liver such as SNHC, focal nodular hyperplasia and hemangioma, eosinophilic cholangitis, idiopathic and others [6].

The standard treatment is surgical resection for malignant biliary stricture. However, balloon dilation or stent insertion has been attempted for benign strictures without the requirement for extensive surgical resection. In addition, deroofing of the cyst, partial hepatectomy including the cyst, percutaneous drainage of the cyst, and the administration of a sclerosing agent can be used as less invasive methods for the treatment of a symptomatic hepatic cyst. However, the differentiation of benign and malignant bile duct strictures is not easy pre-operatively; in cases with a bile duct stricture without a demonstrable mass on CT or US, the distinction cannot be made.

This case represents the smallest SNHC reported to date with symptoms. We could not identify the cyst prior to surgery and had no information on the cause of the left hepatic duct stricture from the imaging studies used in the evaluation, US, ERCP and CT. We performed a left hepatectomy because we could not exclude a malignancy. Because a 2 cm cyst could theoretically be seen on an US or CT, we reviewed the imaging studies retrospectively and found that there was a lesion that could be regarded as cyst on the CT (Figure 1). However, the echogenicity and density of the cyst were so similar to the neighboring ducts, we missed it. In addition, it might have been difficult to accept that such a small cyst could cause a biliary stricture even if it was detected on the pre-operative imaging studies.

If the differential diagnostic markers such as tumor markers, radiological evaluations, cytology by endoscopic approaches, and tissue diagnosis were reliable for the differentiation of benign from malignant bile duct strictures, a less invasive treatment modality might have been appropriate. There have been several reports on the accuracy of the diagnostic markers for the differentiation of benign from malignant bile duct strictures. However, none of these markers are universally accepted for a definitive diagnosis to date [7,8]. This case illustrated that a small SNHC could cause a biliary stricture if in the right location. However, differentiation of benign from malignant disease can be difficult, especially when the imaging studies show no demonstrable mass lesion.

## Conclusions

A very small solitary hepatic cyst might cause hepatic duct stricture if it is located near the hepatic hilum, and should be considered in the differential diagnosis of a hepatic duct stricture.

## Abbreviations

CT: computed tomography; ERCP: endoscopic retrograde cholangiopancreatography; IHBD: intra-hepatic bile ducts; SNHC: solitary non-parasitic hepatic cysts; US: ultrasound.

## Competing interests

The authors declare that they have no competing interests.

## Authors' contributions

TH collected the information and carried out the research. He was the main writer of the manuscript. KL advised, read and approved the final version.

## Consent

Written informed consent was obtained from the patient for publication of this case report and any accompanying images. A copy of the written consent is available for review by the Editor-in-Chief of this journal.
